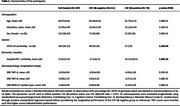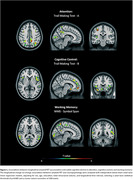# Voxel‐wise longitudinal changes between amyloid PET and executive function subcomponents at the preclinical stage of the Alzheimer's continuum

**DOI:** 10.1002/alz70857_099274

**Published:** 2025-12-24

**Authors:** David López‐Martos, Mahnaz Shekari, Gemma Salvadó, Marc Suárez‐Calvet, Marta Milà‐Alomà, Gwendlyn Kollmorgen, Carolina Minguillón, Henrik Zetterberg, Kaj Blennow, Juan Domingo Gispert, Oriol Grau‐Rivera, Gonzalo Sánchez‐Benavides, for the ALFA study

**Affiliations:** ^1^ Barcelonaβeta Brain Research Center (BBRC), Pasqual Maragall Foundation, Barcelona, Spain; ^2^ Hospital del Mar Research Institute (IMIM), Barcelona, Spain; ^3^ Clinical Memory Research Unit, Department of Clinical Sciences, Lund University, Lund, Sweden; ^4^ Centro de Investigación Biomédica en Red de Fragilidad y Envejecimiento Saludable (CIBERFES), Madrid, Spain; ^5^ Hospital del Mar Research Institute, Barcelona, Barcelona, Spain; ^6^ Servei de Neurologia, Hospital del Mar, Barcelona, Spain; ^7^ Department of Radiology and Biomedical Imaging, University of California, San Francisco, San Francisco, CA, USA; ^8^ Department of Veterans Affairs Medical Center, Northern California Institute for Research and Education (NCIRE), San Francisco, CA, USA; ^9^ Roche Diagnostics GmbH, Penzberg, Germany; ^10^ Centro de Investigación Biomédica en Red de Fragilidad y Envejecimiento Saludable (CIBERFES), Instituto de Salud Carlos III, Madrid, Spain; ^11^ UK Dementia Research Institute at UCL, London, United Kingdom; ^12^ Department of Neurodegenerative Disease, UCL Institute of Neurology, London, United Kingdom; ^13^ Wisconsin Alzheimer's Disease Research Center, University of Wisconsin School of Medicine and Public Health, University of Wisconsin‐Madison, Madison, WI, USA; ^14^ Department of Psychiatry and Neurochemistry, Institute of Neuroscience and Physiology, The Sahlgrenska Academy, University of Gothenburg, Mölndal, Sweden; ^15^ Hong Kong Center for Neurodegenerative Diseases, Hong Kong, China; ^16^ Clinical Neurochemistry Laboratory, Sahlgrenska University Hospital, Mölndal, Sweden; ^17^ Department of Psychiatry and Neurochemistry, Institute of Neuroscience & Physiology, the Sahlgrenska Academy at the University of Gothenburg, Mölndal, Gothenburg, Sweden; ^18^ Centro de Investigación Biomédica en Red de Fragilidad y Envejecimiento Saludable (CIBERFES), Instituto de Salud Carlos III, Barcelona, Spain

## Abstract

**Background:**

Emerging evidence highlights the vulnerability of Executive Function (EF) in subtle cognitive decline, which may precede the clinical onset of Alzheimer's disease (AD). However, how pathological Amyloid (Aβ) accumulation over time relates to EF neuropsychological change remains poorly understood. Here, we investigated whole‐brain voxel‐wise longitudinal changes in Aβ Positron Emission Tomography (PET) imaging in relationship to longitudinal changes across distinct EF subcomponents—attention, cognitive control, and working memory—in cognitively unimpaired (CU) individuals at risk for AD dementia.

**Method:**

One hundred and eighty‐seven CU individuals (mean age: 60.97, SD: 4.81) from the ALFA+ cohort study were included. Participants underwent Cerebrospinal Fluid (CSF) sampling, longitudinal [^18^F]flutemetamol PET acquisition, and longitudinal neuropsychological assessment at baseline and follow‐up (mean years: 3.21; SD: 0.38). Aβ‐positivity was defined as CSF Aβ42/40 <0.071. Neuropsychological changes in EF were evaluated through standardized regression‐based indices, considering the longitudinal performance of the Aβ‐negative group as reference (Attention: *Trail Making Test ‐ A*, Cognitive Control: *Trail Making Test ‐ B*, Working Memory: *Wechsler Memory Scale‐IV ‐ Symbol Span*). The longitudinal change‐on‐change associations between Aβ accumulation and EF neuropsychological change were analyzed with whole‐brain voxel‐wise linear regression models.

**Result:**

Seventy‐six individuals (40.64%) were Aβ‐positive (Table 1). Subtle cognitive decline in attention (TMT‐A) was primarily associated with parietal Aβ accumulation, particularly in the precuneus and postcentral gyrus, as well as Aβ accumulation in the mid‐cingulate cortex, with additional involvement of occipital regions. Cognitive control (TMT‐B) was primarily associated with frontal Aβ accumulation, particularly in the middle and anterior cingulate cortex, along with the inferior frontal cortex, medial frontal cortex, and orbitofrontal cortex. Working memory (Symbol Span) was primarily associated with parietal and temporal Aβ accumulation, specifically in the precuneus, middle temporal cortex, and superior temporal cortex, with additional involvement of occipital regions (Figure 1).

**Conclusion:**

Subtle cognitive decline across core EF subcomponents—attention, cognitive control, and working memory—was associated with distinct regional patterns of amyloid accumulation in CU individuals. These distinctive change‐on‐change signatures offer insights into the specific brain regions implicated in AD‐related EF decline and underscore the importance of tailored neuropsychological assessments for monitoring subtle cognitive changes in preclinical AD.